# Prevalence and severity of menopause symptoms among perimenopausal and postmenopausal women aged 30-49 years in Gulele sub-city of Addis Ababa, Ethiopia

**DOI:** 10.1186/s12905-017-0484-x

**Published:** 2017-12-08

**Authors:** Engida Yisma, Natnael Eshetu, Stephanie Ly, Berhanu Dessalegn

**Affiliations:** 1 School of Allied Health Sciences, College of Health Sciences, Addis Ababa University, Addis Ababa, Ethiopia; 2 Department of Nursing and Midwifery, College of Health Sciences, Mizan-Tepi University, Mizan, Ethiopia; 3 California Center for Population Research and Department of Community Health Sciences, UCLA Fielding School of Public Health, University of California Los Angeles (UCLA), Los Angeles, CA USA

**Keywords:** Menopause, Reproductive, Aging, Ethiopia

## Abstract

**Background:**

Menopause is a natural phenomenon occurring as women approach middle-age. It is characterized by declining of ovarian function and onset of the last menstrual period and subsequent 12-month cessation of menstruation. Due to a growing aging population and longer life expectancies, sub-Saharan African women will spend a significant portion of their lives in postmenopause. Menopausal symptom research has been primarily conducted on Caucasian women from high-income countries. Understanding menopause symptom prevalence and perceptions among diverse women in Ethiopia will better inform reproductive health care.

**Methods:**

We conducted a multi-stage, cross-sectional study on 226 perimenopausal and postmenopausal women aged 30-49 years in Gulele sub-city of Addis Ababa, Ethiopia. Data on socio-demographic characteristics, menopausal status and an 11-item Menopause Rating Scale (MRS) were collected using interviewer-administered questionnaire. Statistical analyses consisted of descriptive data and chi-squared analyses.

**Results:**

Study participants were 40.4 ± 5.9 years, on average, with the majority married, educated at secondary school level, and comprised different ethnicities and wealth tertiles. The most prevalent types of menopausal symptoms reported from the MRS were from somatic subscale (65.9%) while psychological (46.0%) and urogenital subscale symptoms (30.5%) were also prevalent. The most commonly reported individual symptoms were: hot flushes (65.9% (95% CI: 59.4%–72.1%)), difficulty falling asleep (49.6% (95% CI: 42.9%-56.3%)), depressive mood (46.0% (95% CI: 39.4%-52.8%)), irritability (45.1% (95% CI: 38.5%-51.9%)), and anxiety (39.8% (95% CI: 33.4%-46.5%)). Each of the somatic, psychological, and urogenital MRS subscale scores were higher among postmenopausal women compared to perimenopausal women. Women self-reported differing severity levels of symptoms with high severity reported in 8.4% of total MRS, 1.3% of somatic, 10.6% of psychological, and 8.4% of urogenital scales.

**Conclusions:**

Our study provides the first presentation of menopausal symptoms among perimenopausal and postmenopausal women in the Gulele sub-city of Addis Ababa, Ethiopia. We capture the prevalence of menopausal symptoms experienced as well as self-rated severity through the MRS. Our findings reflect menopausal symptoms in our specific study population and have been found to be consistent with previous international research.

**Electronic supplementary material:**

The online version of this article (10.1186/s12905-017-0484-x) contains supplementary material, which is available to authorized users.

## Background

The World Health Organization (WHO) projects a rise in the proportion of adults over age 60 in sub-Saharan Africa from 46 million people in 2015 to 157 million by 2050 [1]. Similarly, sub-Saharan African women can expect an increased life expectancy at 76 years, on average. The prevalence of women experiencing menopause, commonly between the ages of 45 and 55, will also markedly increase. This population poses unique considerations given that sub-Saharan African women will spend a larger proportion of their lives in postmenopause. In Ethiopia alone, the 2007 National Census enumerated 9.7 million women aged 30 or older with 2.1 million in the menopausal age range of 45 to 55 years [2].

Menopause is a natural phenomenon signaling the reduction of ovarian function and onset of the last menstrual period and is generally diagnosed in retrospect since confirmation occurs only after a 12-month cessation of menstrual periods [3]. Perimenopause is defined as the transition prior to the last menstrual cycle, when a woman may experience variable or irregular menstrual cycles and hormonal fluctuations, and the 12 months after the final menstrual period. Premenopause is the stage after menarche but before entering menopausal stages with normal fertility function during this phase [4, 5]. Postmenopause is defined as the stage beginning 12 months after the last menstrual cycle [6].

As a woman enters perimenopause, her individual experiences may coincide with role changes and symptom experiences. Symptoms attributed to menopause vary between individuals and cultures, which has been attributed to general aging, menopausal fluctuations, or socially constructed phenomena [7]. The most commonly reported symptoms among women in high-income countries are vasomotor symptoms including hot flushes, vaginal dryness, insomnia, fatigue, and joint pain [8–10]. Several studies conducted among Nigerian women reported menopausal symptoms of joint and bone pain, hot flush, insomnia, anxiety, urinary symptoms, fatigue, and dyspareunia [11–13]. A study conducted among menopausal Ghanaian women reported tiredness, sleeplessness, palpitations, weight gain, hot flushes, and irritability as their common symptoms [14]. The majority of menopause research has been conducted primarily on Caucasian women from high-income countries, which may influence self-reported symptom in international studies. Inconsistent reports of hot flushes, sexual dysfunction, and depression were found across different ethnicities and countries [15]. The literature has been unclear about associations between menopausal experience with ethnicity, country of origin, or social construction. Furthermore, there is a paucity of research has been conducted on women living in low- and middle-income countries.

In Ethiopia, no known studies investigating the experience of perimenopause has been published. In the present study, we aim to add contextual knowledge to an urban Ethiopian population of women undergoing menopausal transition. We specifically explore prevalence and severity of symptoms among perimenopausal and postmenopausal women aged 30-49 in Gulele sub-city of Addis Ababa, Ethiopia. The restricted age range represents potential variation in entry into menopause, which mean age of menopause among urban Ethiopian women is limited. While the average age of menopause is 51 years globally, the average age varies across geographic regions and is unknown in Ethiopia. To the authors’ knowledge, this article will be the first publication in this sub-population and findings have implications to improve and expand upon menopausal health.

## Methods

### Study design and participants

The study utilized a multi-stage, cross-sectional study design conducted from April 26, 2015 to May 10, 2015 in the Gulele sub-city. Gulele is one of the ten sub-cities comprising Addis Ababa, Ethiopia. The study was designed to recruit women, defined at age 30-49 years to understand their perceptions and attitudes as they approach their last menstrual period. The following eligibility criteria were applied at recruitment: females, current resident in Gulele sub-city, and aged 30-49 years. Study protocols were ethically approved by an ethical clearance committee of the Department of Nursing and Midwifery of Addis Ababa University. Community-level approvals were given by the Addis Ababa city Administration Health Bureau and Gulele Health Office. Informed verbal and written consents were obtained for each participant.

A multistage, clustered random sampling design was applied. In the first stage, three out of 10 woredas (districts) in the Gulele sub-city were selected by simple random sampling. In the second stage, a total of 8 ketenas (villages) were randomly selected from the three woredas (secondary sampling unit) by simple random sampling. In the third stage, households were selected in each ketena using systematic random sampling. Finally, in the fourth stage, all eligible women in the selected households were recruited for the study until the proportionally allocated sample size was reached.

A total of 599 women aged 30-49 years were identified as eligible for an interview and 588 women consented and participated. In this paper, we included a sub-study of the total participants. We specifically restricted our analysis to women that experienced perimenopause or postmenopause and excluded those premenopause. This sub-analysis allowed us to focus on women actively or recently experiencing menopause. Our analysis consisted of 226 perimenopausal and postmenopausal women aged 30-49 years in Gulele sub-city of Addis Ababa, Ethiopia.

### Sample size calculation

The study sample size was estimated using a single population proportion formula, which was calculated with the following assumptions: 95% confidence level, 5% margin of error, and 10.4% expected prevalence of loss of interest in sex as a reported menopause symptom among perimenopausal women (based on a similar study finding among perimenopausal women in Singapore) [16]. Given these assumptions, the required sample size was determined to be 143. Considering a conservative design effect of 1.5 for complex sampling and a non-response rate of 5%, the final estimate was 226. Since the larger study included other research objectives, the overall study sample size recruited 599 women aged 30-49. The results in this article were based on the sub-sample of 226 perimenopausal and postmenopausal women.

### Data collection instruments and procedures

Data were collected using a pre-tested, interviewer-administered questionnaire, which was initially prepared in English and then translated into Amharic (Additional file 1). The study questionnaire was adapted from Ethiopia Demographic and Health Survey 2011 questionnaire [17], a previous menopausal study, and items from relevant literature reviews [18, 19]. Socio-demographic characteristics, menopausal status, and an 11-item Menopause Rating Scale (MRS) were collected from participants [20]. The MRS is a self-reported subjective scale that has been used in different international populations and validated in clinical and epidemiological studies on menopause symptoms [21]. The MRS is organized with multiple items ranked from 0 (not present) to 4 (very severe) on three main areas: (a) *Somatic*: hot flushes, heart discomfort, sleeping problems, and muscle or joint problems; (b) *Psychological*: depressive mood, irritability, anxiety, and physical or mental exhaustion; and (c) *Urogenital*: sexual issues, bladder problems, and dryness of the vagina. Scores are calculated for individual women as sums of each graded item, each subscale, and overall total. Scores equal to or above 9 (somatic), 7 (psychological), 4 (urogenital), and 17 (total) were defined as severe [21, 22].

The questionnaire was tested for comprehension among women aged 30-49 years (*n* = 29) recruited from a nearby area outside of the study population and subsequent revisions were incorporated. Data collection was facilitated by government community health extension workers from the local area and one of the investigators (NE) as a supervisor. In Ethiopia, health extension workers are salaried female community health workers with secondary-level school education and have completed a one-year training in basic health service delivery. They are selected from the community that they serve, to ensure local familiarity, and work at 75% time [23]. The health extension workers received a day-long training on interviewer-based data collection. Each consented participant was asked whether they had experienced the 11 menopausal symptoms shown in the MRS in the last one month (30 days).

Due to the lack of an appropriate monthly income scale to rank economic status, we decided to rank participants’ monthly income based on equally numbered percentile groups. We classified income levels into tertiles, each representing 33.33 percentile levels. Each participant was designated at the lowest, medium, or highest income levels.

### Menopausal status definitions

Menopausal status definitions were adapted from classifications defined by the World Health Organization (WHO) [24]. Perimenopausal status included women experiencing irregular menses within the last 12 months or an absence of menstrual bleeding for more than 3 months but less than 12 months. Postmenopausal status included women that had spontaneously terminated menstrual bleeding for at least 12 months. Since the study aimed to investigate the prevalence and severity of menopause symptoms, women with iatrogenic menopause (*n* = 9), defined as terminated menstruation after a surgical and/or medical intervention (e.g. chemotherapy or radiotherapy to the ovaries or hysterectomy), were considered postmenopausal [19, 24, 25].

### Statistical analysis

The data were entered using the software Epi Info version 7.1.4.0 (CDC, Atlanta, GA, USA). Data were then exported to SPSS version 23.0 (IBM, Armonk, NY, USA) for further processing. Variable recoding and transformations were completed before the final data analysis. Descriptive analyses were performed on all data collected. Chi-Square (χ^2^) tests of independence were used to examine the association between menopausal status of women and their background information.

## Results

A total of 226 perimenopausal and postmenopausal women aged 30-49 years were included in the study. Among the sampled participants, the mean age was 38.9 years (SD ±5.4) for perimenopausal and 43.5 years (SD ±5.8) for postmenopausal women. Socio-demographic factors of participants included: 158 (69.9%) married, 79 (35.0%) completed secondary (high school) education, 94 (41.6%) full-time homemakers, 44 (19.5%) private sector employees, 40 (17.7%) civil servants, 161 (71.2%) Orthodox Christian, 41 (18.1%) Muslim, and 17 (7.5%) Protestant. Details of the background characteristics of study participants by menopause status are given in Table 1.

**Table 1:** Characteristics of the study participants by menopause status, Gulele sub-city of Addis Ababa, Ethiopia

	All (*n* = 226)	Menopausal status		*P*
		Perimenopause (*n* = 151)	Postmenopause (*n* = 75)	
Age				<0.001
30-34 35-39 40-44 45-49 Mean (±SD)	48 (21.2) 47 (20.8) 44 (19.5) 87 (38.5) 40.4 (±5.9)	38 (25.5) 41 (27.2) 35 (23.2) 37 (24.5) 38.9 (±5.4)	10 (13.3) 6 (8.0) 9 (12.0) 50 (66.7) 43.5 (±5.8)	
Education				<0.001
None Primary Secondary College/Uni	53 (23.5) 62 (27.4) 79 (35.0) 32 (14.2)	19 (12.6) 43 (28.5) 60 (39.7) 29 (19.2)	34 (45.3) 19 (25.3) 19 (25.3) 3 (4.0)	
Ethnicity^a^				>0.05
Amhara Oromo Tigre Gurage Others	120 (53.1) 58 (25.7) 28 (12.4) 14 (6.2) 6 (2.7)	79 (52.3) 37 (24.5) 23 (15.2) 9 (6.0) 3 (2.0)	41 (54.7) 21 (28.0) 5 (6.7) 5 (6.7) 3 (4.0)	
Marital status				<0.05
Married Single Widowed Divorced	158 (69.9) 25 (11.1) 30 (13.3) 13 (5.8)	109 (72.2) 20 (13.3) 13 (8.6) 9 (6.0)	49 (65.3) 5 (6.7) 17 (22.7) 4 (5.3)	
Religion				>0.05
Orthodox Muslim Protestant Others	161 (71.2) 41 (18.1) 17 (7.5) 7 (3.1)	111 (73.5) 27 (17.9) 10 (6.6) 3 (2.0)	50 (66.7) 14 (18.7) 7 (9.3) 4 (5.3)	
Occupation				<0.001
Housewife/Homemaker Private employee Civil servant Merchant NGO worker Others	94 (41.6) 44 (19.5) 40 (17.7) 28 (12.4) 11(4.9) 9 (4.0)	47 (31.1) 33 (21.9) 34 (22.5) 23 (15.2) 11 (7.3) 3 (2.0)	47 (62.7) 11 (14.7) 6 (8.0) 5 (6.7) 0 (0.0) 6 (8.0)	
Monthly income				
(wealth tertiles)				<0.001
Lowest Medium Highest	95(42.0) 58 (25.7) 73 (32.3)	45 (29.8) 41 (27.2) 65 (43.0)	50 (66.7) 17 (22.7) 8 (10.7)	

^a^In Ethiopia, Oromo comprises the largest proportion of ethnic groups followed by Amhara, Tigre and others like Gurage

### Menstrual history and menopausal status of the study participants

The majority of participants (66.8%) knew their age at menarche with 129 women (85.4%) reported menarche at age 13 years or older while 22 women (14.6%) were less than 13 years old. When assessing current menopausal statuses of participants, 151 (66.8%) women were perimenopausal and 75 (33.2%) were postmenopausal. Of the total 75 postmenopausal women included in our current study, 9 women had hysterectomy. Age at Last Menstrual Period (LMP) was collected for postmenopausal women only and the mean age at LMP for postmenopausal women was 42.6 (SD ±4.2 years) while the average duration of menopause (number of years since LMP) for these women was 2.6 (SD ± 1.8 years).

### Prevalence of menopausal symptoms

Overall, the five most commonly reported menopause symptoms experienced in the last month as assessed by the MRS included: Hot flushes (65.9% (95% confidence interval [CI]: 59.4%–72.1%)), difficulty falling asleep (49.6% (95% CI: 42.9%-56.3%)), depressive mood (46.0% (95% CI: 39.4%-52.8%)), irritability (45.1% (95% CI: 38.5%-51.9%)) and anxiety (39.8% (95% CI: 33.4%-46.5%)). The least prevalent menopause symptoms experienced by women also included: heart discomfort (22.1%), bladder problems (26.1%) and sexual problems (27.0%).

### Prevalence of menopausal symptoms according to menopausal status of women

The prevalence and specific symptoms experienced by each woman differed based on their menopausal status. Postmenopausal women more commonly reported experiencing all three menopause symptom subscale categories (somatic, psychological and urogenital) when compared to perimenopausal women.

Hot flush was the most prevalent somatic subscale symptom reported by both perimenopausal and postmenopausal women in 57.0% and 84.0% of cases, respectively. For psychological subscale symptoms, perimenopausal women most commonly described depressive mood (36.4%) symptoms while both depressive mood and irritability (65.3%) were the most prevalent symptoms experienced by postmenopausal women. For postmenopausal women, dryness of the vagina (64.0%) was the most commonly reported urogenital subscale symptom complaint whereas sexual problems (15.2%) was the most prevalent urogenital symptom reported by perimenopausal women (Table 2).

**Table 2:** Proportion of menopausal symptoms among participants according to menopausal status

Subscale (menopausal symptoms)	All (*n* = 226)	Perimenopausal (*n* = 151)	Postmenopausal (*n* = 75)
Somatic			
Hot flushes Heart discomfort Difficulty of falling asleep Muscle and joint problems	149 (65.9) 50 (22.1) 112 (49.6) 73 (32.3)	86(57.0) 22(14.6) 59 (39.1) 28 (18.5)	63(84.0) 28(37.3) 53(70.7) 45(60.0)
Psychological			
Depressive mood Irritability Anxiety Physical and sexual exhaustion	104 (46.0) 102 (45.1) 90 (39.8) 79 (35.0)	55(36.4) 53(35.1) 46(30.5) 36 (23.8)	49(65.3) 49(65.3) 44 (58.7) 43 (57.3)
Urogenital			
Sexual problems Bladder problems Dryness of the vagina	61 (27.0) 59 (26.1) 69 (30.5)	23 (15.2) 19(12.6) 21 (13.9)	38 (50.7) 40 (53.3) 48 (64.0)

### Severity of menopausal symptoms

The severity of menopause symptoms was investigated based on the MRS scoring system. About 8.4% of women presented total MRS scores above 17, which is defined as severe. The severity of menopause symptoms by subscale was also investigated. About 10.6% of women scored in the severe range on psychological subscale, 8.4% scored as severe on somatic, and 1.3% on the urogenital subscale (see Table 3).

**Table 3:** Frequency of severe MRS score per each subscale and overall symptoms score of menopause symptoms among perimenopausal and postmenopausal women aged 30-49 years in Gulele, Addis Ababa, Ethiopia

Menopause symptoms	Frequency (*n* = 226)	Percent
Somatic symptoms	3	1.3
Psychological symptoms	24	10.6
Urogenital symptoms	19	8.4
Overall symptoms	19	8.4

Severity was also assessed for specific menopausal symptoms as detailed in each subscale of the MRS. Table 4 shows the prevalence of self-rated severity of menopause symptoms from mild to very severe. A small proportion of women (4.0% (95% CI: 1.8%-7.4%)) rated hot flush as severe while 35.0% (95% CI: 28.8%-41.6%) of women rated the symptom as mild.

**Table 4:** Prevalence of self-rated severity of menopausal symptoms among women aged 30-49 in Gulele, Addis Ababa, Ethiopia

Symptoms	Mild % (95% CI)	Moderate % (95% CI)	Severe % (95% CI)	Very severe % (95% CI)
Hot flushes	34.96 (28.8-41.6)	26.55 (20.9-32.8)	3.98 (1.8-7.4)	0.4 (0.01-2.4)
Sleep problems	35.40 (29.17-42.01)	11.50 (7.65-16.40)	2.21 (0.72-5.09)	0.44 (0.01-2.44)
Depressive mood	34.51 (28.33-41.10)	10.18 (6.56-14.88)	1.33 (0.27-3.83)	–
Irritability	30.53(24.60-36.98)	11.95 (8.02-16.90)	2.65 (0.98-5.69)	–
Anxiety	27.43 (21.73-33.74)	10.18 (6.56-14.88)	2.21 (0.72-5.09)	–
Physical and mental exhaustion	24.34 (18.89-30.47)	8.41 (5.14-12.82)	2.21 (0.72-5.09)	–
Joint and muscular discomfort	16.81 (12.18-22.34)	13.27 (9.14-18.40)	2.21 (0.72-5.09)	–
Dryness of vagina	23.01 (17.69-29.05)	5.31 (2.77-9.09)	2.21 (0.72-5.09)	–
Sexual problems	19.47 (14.52-25.24)	5.31 (2.77-9.09)	2.21 (0.72-5.09)	–
Bladder problems	17.26 (12.57-22.83)	6.19 (3.43-10.18)	2.65 (0.98-5.69)	–
Heart discomfort	14.69 (10.27-19.89)	6.19 (3.43-10.18)	1.33 (0.27-3.83)	–

## Discussion

As the demographic shift within Ethiopia and the sub-Saharan African region moves towards an aging population and longer life expectancies, the importance of understanding health needs among older adults is relevant. While research on women in Ethiopia is being conducted, the focus has been on maternal and adolescent health but little attention has been paid to menopausal health. The Menopause Rating Scale is a globally recognized instrument measuring three symptomatic menopause categories with no existing publications gathering data on women from Addis Ababa, Ethiopia. In this study, we applied the first recorded use of the MRS in an urban Ethiopian population.

Women selected for participation in our study (*n* = 226) had a mean age of 40.4 years, were married, educated to secondary school level, identified with Amharan and Oromian ethnicities, and practiced Orthodox Christianity. The majority of women were aged 13 or older at menarche, which is consistent with the average age at menarche at 13.5 years among women in a review of 67 countries [26]. All participants included in this sub-analysis were in perimenopausal and postmenopausal stages with mean age at 38.9 and 43.5 years, respectively. Age at the last menstrual period for women in postmenopause was 42.6 years and the average age of duration of menopause (number of years since LMP) for postmenopausal women was 2.6 years. Since this study was the first to collect menopausal data in Addis Ababa, Ethiopia, the mean ages at menarche and menopause have yet to be reported nationwide to generalize ages to the larger population of Ethiopian women.

Ethiopian menopausal women experienced somatic symptoms (65.9%) most prevalently, followed by psychological (46.0%), and least prevalently, urogenital symptoms (30.5%). The five most commonly reported specific symptoms were: hot flushes (65.9%), difficulty falling asleep (50.0%), depressive moods (46.0%), irritability (45.1%) and anxiety (39.8%), which is consistent with literature in other diverse populations [13, 27, 28]. When assessing current menopausal status and symptoms, we found that postmenopausal women experienced higher proportions of nearly every type of menopausal symptoms while perimenopausal women reported lower frequency of symptoms. Among all participants, only 8.4% of women presented total MRS as severe. When considering the most commonly reported symptoms including hot flushes, sleep problems, depressive mood, irritability, anxiety for severity, most women reported their symptoms as mild.

Despite the growing population of aging women globally, few studies have been conducted on menopause in low- and middle-income countries. The sub-Saharan population found to have menopausal articles focused in Nigeria. Our research, similar the Nigerian studies, found high proportions of self-reported bone and joint pain, hot flushes, and physical and mental exhaustion [11–13, 27]. Nigerian women tended to report symptoms as mild with only 8.6% of participants rating symptoms as severe, which is also consistent with our severe prevalence rate [13]. A recent Nigerian study found high prevalence of additional symptoms such as insomnia, headache, forgetfulness and palpitations [12]. However, different symptom types were reported in different proportions as compared to the Nigerian studies.

In this study, menopausal vasomotor symptoms, such as hot flushes and night sweats, were the most commonly reported symptoms. The potential to manage vasomotor symptoms using hormone therapy like low-dose oral estradiol or non-hormonal alternatives such as venlafaxine and paroxetine exists [29]. These treatments, in addition to greater awareness of menopausal symptoms and experiences, could form an open dialogue and approach to menopausal management in Addis Ababa.

Our current study has opportunities for expansion on more comprehensive background information, such as detailed medical history, family history, reproductive health experiences, and family member experiences. Additionally, anthropometric measures could be added such as height and weight to explore findings from three separate studies where researchers found that women with a higher body mass index reported more vasomotor symptoms [30–32]. Other studies have evidenced high levels of physical activity with lower frequency of vasomotor symptoms [33]. Lifestyle factors like smoking and alcohol use have also been linked to hot flushes and earlier menopausal age [33–35]. The onset of menopause and experience of symptoms are also influenced by reproductive factors such as women with low number of births no children, spent limited or no time breastfeeding, or had low usage of oral contraceptives reported an earlier onset of menopause [33]. Thus, we believe that future research potential on menopausal Ethiopian women could have far-reaching novel areas.

The current study has notable limitations. First, the study assessed only the prevalence and severity of menopause symptoms among women aged 30-49 years in the Gulele sub-city of Addis Ababa, Ethiopia. Our findings are not generalizable to the overall Ethiopian female population nor Addis Ababa. Second, the original study had an age restriction at 49 years to capture the perimenopausal stage but lacks samples of women further in postmenopause. Third, the study did not collect data on hormonal therapy use and/or other options for treatment of menopause symptoms. Finally, our study was a cross-sectional design, limiting ability to infer causality and confounding. Longitudinal studies addressing a larger population of Ethiopian women to assess menopause symptom experience and possible risk factors should be established. Given limitations in research infrastructure, our study conducted the most available research on this special population.

## Conclusions

The prevalence and severity of menopause symptoms among perimenopausal and postmenopausal women aged 30-49 years residing in Gulele was assessed using the Menopause Rating Scale (MRS). The most commonly reported individual symptoms were: hot flushes, difficulty falling asleep, depressive moods, irritability and anxiety. The somatic, psychological, and urogenital subscale symptoms of menopause were higher among postmenopausal women compared to perimenopausal women. Women reported psychological menopause symptoms as the most severe experience.

Our initial results provide an opportunity for expansion into further studies to enumerate the true prevalence of menopause and menopausal symptoms in Ethiopia. The MRS instrument could be applied to different urban and rural populations. Future work should be conducted to better understand the unmet health needs of Ethiopian women entering or experiencing menopause.

## Additional file

Additional file 1: The study questionnaire in both English and Amharic languages. It consists of comprehensive information regarding the participants’ socio-demographic characteristics, menopausal status, menopause knowledge and attitude, and an 11-item Menopause Rating Scale. (PDF 365 kb)

## Abbreviations

CDC: Centers for Disease Control and Prevention
CI: Confidence Interval
LMP: Last Menstrual Period
MRS: Menopause Rating Scale
WHO: World Health Organization
