# Supplementary material for: Prevalence and severity of menopause symptoms among perimenopausal and postmenopausal women aged 30-49 years in Gulele sub-city of Addis Ababa, Ethiopia
Source: BMC Womens Health. 2017 Dec 8;17:124. doi: 10.1186/s12905-017-0484-x (PMC5721600; doi:10.1186/s12905-017-0484-x)
Supplement: Additional file 1: — The study questionnaire in both English and Amharic languages. It consists of comprehensive information regarding the participants’ socio-demographic characteristics, menopausal status, menopause knowledge and attitude, and an 11-item Menopause Rating Scale. (PDF 365 kb) [file 12905_2017_484_MOESM1_ESM.pdf]

I: English version questionnaire

Consent form

Hello! My Name is ________________. I am a member of a research team in Addis Ababa University, College of Health Science, School of Allied Health Science, Department of Nursing and Midwifery. I would like to inform you that you and I would have a short discussion concerning our study. Before we go to our discussion, I will request you to listen carefully to what I am going to read to you about the purpose and general information of the study, and you will tell me whether you agree or disagree to participate in the study.

Read the following paragraph for the participant.

The purpose of we are conducting this study is to assess knowledge and attitude of women towards menopause among women aged 30-49 years in Gulele sub-city of Addis Ababa, Ethiopia. We are kindly requesting you for a little of your time, about 20 minutes, to be involved in this study. In the end, it is hoped that the information you give us could help to design appropriate reproductive health services for menopause women. We would like to assure you that your name will not be used and your responses, to any of the questions, will not be given to anyone else and no reports of the study will ever identify you. If the study is published, only information about the total group will appear. The interview is on voluntary. Your participation, non-participation or refusal to respond to the questions will have no effect now or in the future on services that you or any member of your family may receive from any service providers. Only volunteers will participate in this study.

Are you willing to participate in this study?

[ ] Yes [ ] No Signature_______________

Thank you!

Part I: Sociodemographic characteristics

Question		Response	Code
1.	Questionnaire code	-------------------	C0
2.	Woreda	-------------------	C1
3.	Ketena	-------------------	C2
4.	House number	-------------------	C3
5.	Age	------------ (in year)	C5
6.	Educational status	1. Unable to read and write<br>2. Able to read and write<br>3. 1-4 grade<br>4. 5-8 grade<br>5. 9-10 grade<br>6. 11-12 grade<br>7. College/university student<br>8. Post graduate degree<br>88. Refused	C6
7.	Ethnicity	1. Amhara<br>2. Oromo<br>3. Tigre<br>4. Gurage<br>5. Other (specify) -------------<br>88. Refused	C7
8.	Marital status	1. Single<br>2. Married<br>3. Divorced<br>4. Widowed<br>88. Refused	C8
9.	Religion	1. Muslim<br>2. Orthodox<br>3. Protestant<br>4. Catholic<br>5. Other (specify)------------<br>88. Refused	C9
10.	Occupation	1. House wife<br>2. Merchant<br>3. Civil servant<br>4. Daily laborer<br>5. Unemployed<br>6. NGO employee<br>7. Unpaid employee<br>8. Employed in private sector<br>9. Student<br>10. Other (specify):-------------<br>88. Refused	C10
11.	Taking the past year, can you tell me what the average earnings of the household have been within a month?	---------------- ETB/month<br>88. Refused	C12

Part II: reproductive health history

Question		Response	Code
1.	When your first menstrual period started?	1. Under 13 years old<br>2. 13 years old<br>**3.** Older than 13 years old<br>**4.** I didn't know	T0
2.	Do you have children?	1. Yes<br>**2.** No If No, go to T3	T1
3.	If yes, what is the number of your children? (Enter number)	No └─┴─┘	T2
4.	Did you have any miscarriages, abortions or stillbirths?	1. Yes<br>**2.** No If No, go to T5	T3
5.	If yes, how many miscarriages, abortions or stillbirths you had experienced?	No └─┴─┘	T4
6.	Do you use contraceptive?	1. Yes<br>**2.** No If No, go to T7	T5
7.	If Yes, Which Method Are You Using?	1. Female Sterilization<br>2. Injectable<br>3. Male Condom<br>4. Female Condom<br>5. Diaphragm/Foam/Jelly<br>6. Standard Days Method<br>7. Oral pills<br>8. Intra-uterine device<br>9. Other (specify) ----------------<br>88. Refused	T6
8.	Are you pregnant now?	1. Yes If yes , go to T10<br>2. No<br>3. Unsure	T7
9.	Are you breastfeeding now?	1. Yes<br>2. No	T8
10.	Do you have an operation to avoid having any more children?	1. Yes<br>2. No	T9
11.	Do you currently perform strenuous exercise?	1. Yes<br>2. No	T10
12.	When was your last menstrual period?	1. Days Ago-----If yes , go to T15<br>2. Weeks Ago---If yes , go to T15<br>3. Months Ago--If yes , go to T15<br>4. Before Last Birth<br>5. 12 months ago<br>6. Has Had Hysterectomy<br>**7.** Never Menstruated	T11
13.	How old were you at your last menstrual period? (ask this question for woman in menopause)	---------------(age in years)	T12
14.	Do you medical reasons for stoppage of your menstrual period?	1. Yes<br>2. No If No, go to T15	T13

15.	Mention your medical condition	-------------------------	T14
16.	How would you describe your current menstrual status?	1. Regular menstrual periods in the last 3 Months. **If yes, go to A0**<br>2. Irregular menstrual periods in the last 12 months<br>3. No menstrual periods in the last 12 months or longer	T15
17.	Which of the following symptoms apply to you at in the past one month? Please, mark the appropriate box for each symptom. For symptoms that do not apply, please mark 'none'.		

			Response					
	**Symptoms**	**Score =**	**None-mild-moderate-severe-very severe**<br>**I --------I---------- I ------------I --------------I**<br>**0**	**1**	**2**	**3**	**4**	
	Hot flushes, sweating		O	O	O	O	O	T16a
	Heart discomfort		O	O	O	O	O	T16b
	Sleep problems		O	O	O	O	O	T16c
	Depressive mood		O	O	O	O	O	T16d
	Irritability		O	O	O	O	O	T16e
	Anxiety		O	O	O	O	O	T16f
	Physical and mental exhaustion		O	O	O	O	O	T16g
	Sexual problems		O	O	O	O	O	T16h
	Bladder problems		O	O	O	O	O	T16i
	Dryness of vagina		O	O	O	O	O	T16j
	Joint and muscular discomfort		O	O	O	O	O	T16k
18.	Total number symptoms apply to you at this time?		No └──┴──┘					T17

Part III: Knowledge on menopause

Now, I am going to ask you some questions about your various concepts of menopause phenomenon.			
Question		Response	Code
1.	Do you think that at the time of menopause, menstruation stops suddenly?	1. Yes<br>2. No	A0
2.	Do you know that women become menopausal at the age of 48-55 years?	1. Yes<br>2. No	A1
3.	Do you know that hereditary background affects the time of menopause occurrence?	1. Yes<br>2. No	A2
4.	Do you know that menopause occurs in women due to increasing sexual hormones?	1. Yes<br>2. No	A3
5.	Do you know that thin people become menopause sooner?	1. Yes<br>2. No	A4
6.	Do you know that most of the women experience menstruation disorder before menopause occurrence?	1. Yes<br>2. No	A5
7.	Do you know that most of the women experience hot flashes in the menopause period?	1. Yes<br>2. No	A6
8.	Do you think that menopause in women decreases genital infection?	1. Yes<br>2. No	A7
9.	Do you believe that menopause in women increases weight and obesity?	1. Yes<br>2. No	A8
10.	Do you feel that menopause symptoms are preventable and curable?	1. Yes<br>2. No	A9
11.	Do you think that menopause decreases Cardiovascular diseases in women?	1. Yes<br>2. No	A10
12.	Do you know that menopause increases osteomalacia in women?	1. Yes<br>2. No	A11
13.	Do you know that menopause causes dryness and skin shrivel in women?	1. Yes<br>2. No	A12
14.	Do you think that menopause causes different types of cancer in women?	1. Yes<br>2. No	A13
15.	Do you know that sexualities change in menopaused women?	1. Yes<br>2. No	A14
16.	Do you know that smoking affects the time of menopause occurrence?	1. Yes<br>2. No	A15

17.	Do you know that smoking does not affect the severity of symptoms and complications of menopause?	1. Yes<br>2. No	A16
18.	Do you think that menopause increases extra hair on women's face?	1. Yes<br>2. No	A17
19.	Do you know that menopause causes vaginal dryness and painful sexual intercourse?	1. Yes<br>2. No	A18
20.	Do you know that menopause causes urinary frequency and dysuria?	1. Yes<br>2. No	A19
21.	Do you know that smoking and using alcohol are factor of increasing osteomalacia in women?	1. Yes<br>2. No	A20
22.	Do you know that regular physical activity is effective in preventing osteomalacia in menopausal women?	1. Yes<br>2. No	A21
23.	Do you know that menopause affects the power of concentration and memory of women?	1. Yes<br>2. No	A22
24.	Do you think that the frequency and severity of hot flashes in menopaused women increase by time?	1. Yes<br>2. No	A23
25.	Do you know that the level of stress and depression feeling increase in menopaused women?	1. Yes<br>2. No	A24
26.	Do you believe that during 1 year after complete stop of menstruation, pregnancy prevention is necessary?	1. Yes<br>2. No	A25

Part IV: Attitude on menopause phenomenon

Now, I am going to ask you some questions about your Attitude on menopause phenomenon.			
Question		Response	Code
1.	Menopause is the period of woman's loneliness	1. Completely agree<br>2. Agree<br>3. Disagree<br>4. completely disagree	P0
2.	Menopause is the period of eradicating the problems of menstruation and preventing pregnancy	1. Completely agree<br>2. Agree<br>3. Disagree<br>4. completely disagree	P1
3.	Woman's menopause decreases husbands sexuality	1. Completely agree<br>2. Agree<br>3. Disagree<br>4. completely disagree	P2
4.	Every woman can care for herself through training and necessary tend	1. Completely agree<br>2. Agree<br>3. Disagree<br>4. completely disagree	P3
5.	In the menopause period, interest and attention of woman to her husband decreases	1. Completely agree<br>2. Agree<br>3. Disagree<br>4. completely disagree	P4
6.	Menopause is the beginning of the period of women's disablement	1. Completely agree,<br>2. Agree<br>3. Disagree<br>4. completely disagree	P5
7.	Woman's life in the menopause period is more delightful than before menopause	1. Completely agree,<br>2. Agree<br>3. Disagree<br>4. completely disagree	P6
8.	Menopause decreases the grace of woman's appearance	1. Completely agree,<br>2. Agree<br>3. Disagree<br>4. completely disagree	P7
9.	Menopause is a usual and natural phenomenon in women's life	1. Completely agree,<br>2. Agree<br>3. Disagree	P8

		4. completely disagree	
10.	Menopause is the beginning of another life and second maturity of women	1. Completely agree,<br>2. Agree<br>3. Disagree<br>4. completely disagree	P9

Part V: Factors that affect knowledge and attitude of the women toward menopause

Now, I am going to ask you some questions about various factors that affect knowledge and attitude of the women toward menopause. This includes ease of talking to others about menopause, physical health, emotional health, source of information, number of friends and family member's respondent could talk to about menopause and menopausal status. Let's start with source of information.

Question		Response	Code
1.	Have you ever received information on Menopause?	1. Yes<br>2. No If No, go to Q3	Q0
2.	If yes, from where did you get the information on Menopause?	1. Friend	Q1a
		2. Medical care providers	Q1b
		3. Books, Magazines, Journals	Q1c
		4. Mass media (Radio, TV)	Q1d
		5. Relatives	Q1e
		6. Educational sessions	Q1f
		7. Community Elder	Q1g
		8. Other, (specify) -----------	Q1h
3.	Do you feel that talking to other about menopause is	1. very easy<br>2. easy<br>3. difficult<br>4. Very difficult.	Q2
4.	How many friends you can talk in a very comfortable way about menopause.	Number of friends └─┴─┘	Q3
5.	How many people in your family you can talk in a very comfortable way about menopause	Number of family └─┴─┘	Q4

6.	How do you perceive/feel your current emotional health	1. Very poor<br>2. Poor<br>3. good<br>4. very good<br>5. excellent	Q5
7.	How do you perceive/feel your current physical health	1. Very poor<br>2. Poor<br>3. good<br>4. very good<br>5. excellent	Q6

II: Amharic version questionnaire

ቃለ መጠይቁ ከመጀመሩ በፊት የኢ-መደበኛ የቃል ስምምነት መጠየቂያ ቅጽ

ጤና ይስጥልኝ ስሜ----------------- ይባላል፡፡ የመጣሁት በአዲስ አበባ ዩኒቨርሲቲ የጤና ሳይንስ ኮሌጅ አላይድ የጤና ሳይንስ ትምህርት ቤት የሚድዋይፍና ነርሲንግ ትምህርት ክፍል የተማራማሪዎች ቡድን አባል ነኝ፡፡ በቅድሚያ ስለምሰራዉ ጥናት ትንሽ ላስተዋዉቅዎትና ከዛ በኋላ ለጥናቴ የሚረዳኝ መረጃ ለማግኘት አጭር የዉይይት ጊዜ ይኖረናል፡፡ ዉይይታችንን ከመጀመራችን በፊት ግን አሁን የማነብልሽን፣ ስለጥናቴ አላማና አጠቃላይ ሁኔታ የሚገልጸዉን ጽሁፍ ሳነብልሽ በጥሞና በማዳመጥ በሃሳቡ በጥናቱ ዉስጥ ለመሳተፍ እንደምትስማሚ ወይም እንደማትስማሚ ትገልጭልኛለሽ፡፡

ቀጣዩን ጽሁፍ ለተመረጠች መላሽ ይነበብ፡፡

የዚህ ጥናት አላማ በአዲስ አበባ ከተማ ጉለሌ ክፍለ ከተማ የሚገኙ እድሜያቸዉ ከ30-49 የሆኑ ሴቶች ስለማረጥ ያላቸዉን የእዉቀትና አመለካከት ደረጃ መገምገም ነዉ፡፡ ስለሆነም ካለሽ ጊዜ ላይ በዚህ ጥናት ለመሳተፍ፣ ሀያ ደቂቃ ቆይታ እንዲኖረን ፈቃድሽ እንዲሆን በትህትና እጠይቃለሁ፡፡ ለጥናቱ የምትሰጪዉ መረጃ በአጠቃላይ ስለ የሚያርጡ ሴቶችን በተመለከተ በስነተዋልዶ ጤና ዘርፍ በሚደረገዉ እቅድ ስለሚጠቅም በቅድሚያ እናመሰግናለን፡፡ በዚህ ጥናት ላይ የምትሰጭዉ መረጃ እንዲሁም ስምሽ በሚስጥር የሚጠበቅ ሲሆን በሌላ ሰዉ እጅም ተላልፎ አይሰጥም ከዚህ ጥናት ዉጭም አገልግሎት ላይ አይዉልም፡፡ የዚህ ጥናት ዉጤት ለህትመት ቢበቃ እንኳን በአጠቃላይ ምርምር ስለተደረገበት ቡድን እንጂ የግለሰቦች ስምና መረጃ በፍጹም አይጠቀስም፡፡ ቃለመጠይቁ በፈቃደኝነት ላይ የተመሰረተ ነዉ፡፡ በዚህ ጥናት ላይ መሳተፍሽ፣ለመሳተፍ አለመፍቀድሽ፣ ወይም መቃወምሽ ወደፊት አንቺ ወይም ከቤተሰቦችሽ አንዱ ከአገልግሎት ሰጪ ተቋማት የሚያገኘዉ ወይም የሚያጣዉ ምንም ነገር የለም፡፡ በዚህ ጥናት የሚሳተፉት በፍላጎት ብቻ ነዉ፡፡

በዚህ ጥናት ለመሳተፍ ፈቃደኛ ነሽ?

[ ] ፈቃደኛ ነኝ [ ] ፈቃደኛ አይደለሁም ፊርማ------------------

አመሰግናለው!

ክፍል 1: የጥናቱ ተሳታፊዎች አጠቃላይ ማህበራዊ መረጃዎች

ጥያቄ		መልስ	መለያ
1.	የመጠይቅ መለያ ቁጥር	-------------------	C0
2.	ወረዳ	-------------------	C1
3.	ቀጠና	-------------------	C2
4.	የቤት ቁ.	-------------------	C3
5.	ዕድሜ	------------ በዓመት	C5
6.	የትምህርት ደረጃ	1. ማንበብና መጻፍ የማይችል<br>2. ማንበብና መጻፍ የሚችል<br>3. ከ1-4ኛ ክፍል<br>4. ከ5-8ኛ ክፍል<br>5. ከ9-10 ኛ ክፍል<br>6. ከ11-12 ክፍል<br>7. የኮሌጅ/ዩኒቨርሲቲ ተማሪ<br>8. ማስተርስ (ድህረ መሩቅ)<br>88. መናገር አልፈልግም	C6
7.	ብሔር	1. አማራ<br>2. ኦሮሞ<br>3. ትግሬ<br>4. ጉራጌ<br>5. ሌላ ካለ (ይጠቀስ)----------------<br>88. መናገር አልፈልግም	C7
8.	የጋብቻ ሁኔታ	1. ያላገባ/ች<br>2. ያገባ/ች<br>3. የፈታ/ች<br>4. የሞተችበት/ባት<br>88. መናገር አልፈልግም	C8
9.	ሀይማኖት	1. ኦርቶዶክስ<br>2. ሙስሊም<br>3. ካቶሊክ<br>4. ፕሮቴስታንት<br>5. ሌላ ካለ (ይጠቀስ)----------------<br>88. መናገር አልፈለገችም	C9
10.	ባለፉት **12** ወራት ዉስጥ ከሚከተሉት የትኛዉ የስራ ሁኔታዎን ይገልፃል?	1. የቤት እመቤት<br>2. ነጋዴ<br>3. የመንግስት ሰራተኛ<br>4. የቀን ሰራተኛ<br>5. ስራ ፈላጊ<br>6. መንግስታዊ ያልሆነ ድርጅት/ NGO / ሰራተኛ<br>7. ሳይከፈለዉ/ላት የሚሰራ/ምትሰራ ሰራተኛ<br>8. በግል ስራ የተሰማራች<br>9. ተማሪ<br>10. ሌላ ካለ (ይጠቀስ)----------------<br>88. መናገር አልፈለገችም	C10
11.	በአማካይ ከባለፈዉ ዓመት ተነስተዉ የቤታችሁ ገቢ በወር ስንት እንደሆነ ሊነግሩኝ ይችላሉ?	---------------- ብር/ወር<br>88. መናገር አልፈለገችም	C12

ክፍል 2: ስለ ስነተዋልዶ ጤና መረጃ

ጥያቄ		መልስ	መለያ
1.	ወር አበባ መጀመሪያ ያየሽዉ መቼ ነዉ?	1. ከ 13 አመት በታች<br>2. በ 13 አመትሽ<br>**3.** ከ 13 አመትሽ በኋላ<br>4. አላስታውስም	T0
2.	ልጆች አሉሽ?	3. አዎ<br>**4.** የለኝም ---------- **ወደ T3 እለፊ**	T1
3.	አዎ ከሆነ መልስሽ ስንት ልጆችአሉሽ?	በቁጥር └─┴─┘	T2
4.	ከዚህ ቀደም በርግዝና ወቅት ችግር ደርሶብሽ ያዉቃል(ሞቶ የተወለደ፣ማስወረድ)?	1. አዎ<br>**2.** አይ---------- **ወደ T5 እለፊ**	T3
5.	አዎ ከሆነ ስንት ጊዜ ደርሶ ያዉቃል(ሞቶ የተወለደ፣ማስወረድ)?	በቁጥር └─┴─┘	T4
6.	የወሊድ መቆጣጠሪያ ትጠቀሚያለሽ?	1. አዎ<br>**2.** አይ---------- **ወደ T7 እለፊ**	T5
7.	አዎ ከሆነ ምን አይነት?	1. ዘላቂ/ቆሚ የወሊድ መቆጣጠሪያ<br>2. የሚወጋ<br>3. የወንድ ኮንዶመ<br>4. የሴት ኮንዶም<br>5. ዲያፍራም /ፎም / ጄል<br>6. በክንድ የሚቀበሩ<br>7. በአፍ የሚወሰድ እንክብል<br>8. ማህጸን ዉስጥ የሚቀመጥ<br>9. ሌላ ካለ (ይጠቀስ)----------------<br>10. 88. መናገር አልፈለገችም	T6
8.	በአሁን ሰአት ነፍሰጡር ነሽ?	1. አዎ---------- **ወደ T10 እለፊ**<br>2. አይ<br>3. እርግጠኛ አደለሁም	T7
9.	በአሁን ሰአት ጡት ታጠቢያለሽ?	1. አዎ<br>2. አይ	T8
10.	አሁን ካለሽ በላይ ልጅ እንዳይኖርሽ ብለሽ ኦፐራሲዮን አድርገሽ ታዉቂያለሽ?	1. አዎ<br>2. አይ	T9
11.	በአሁን ሰአት ከፍተኛ የአካል እንቅስቃሴ ታደርጊያለሽ?	1. አዎ<br>2. አይ	T10
12.	ለመጨረሻ ጊዜ የወርአበባ ያየሽዉ መቼ ነዉ?	1. ከቀናት በፊት------------**ወደ T15 እለፊ**<br>2. ከሳምንታት በፊት-------**ወደ T15 እለፊ**<br>3. ከወራት በፊት---------- **ወደ T15 እለፊ**<br>4. ከ12 ወር በፊተረ<br>5. ማኅጸኔን ካስወጣው በዋላ<br>6. ከመጨረሻዉ ወሊድ በፊት<br>**7.** መጥቶብኝ አያዉቅም	T11
13.	ላረጠች ሴት የሚቀርብ ጥያቄ) ለመጨረሻ ጊዜ የወርአበባ ያየሽዉ በስንት አመትሽ ነዉ?	---------------(እድሜ በቁጥር ይገለጽ)	T12
14.	ለወር አበባሽ መቆም የህክምና/ከጤና ጋር የጠያያዘ ምክንያት አለሽ?	1. አዎ<br>2. አይ ---------- **ወደ T15 እለፊ**	T13
15.	የህክምና/ከጤና ምክንያቱን ይጠቀስ	-------------------------	T14

16.	አሁን ያለሽበትን የወር አበባ ሁኔታ እንዴት ትገልጪዋለሽ?	1. ላለፉት ሶስት ወራት ሳይዛባ የወር አበባዬ መጥቷል---------- **ወደ A0 እለፊ**<br>2. ላለፉት አስራሁለት ወራት በተዛባ ሁኔታ የወር አበባዬ መጥቷል<br>3. ላለፉት አስራሁለት ወራት የወር አበባዬ አልመጣም						T15
17.	ከሚከተሉት ምልክቶች ዉስጥ የትኞቹ በአንቺ ላይ ታይቷል . ከተሰጡት ሳጥኖች ዉስጥ ምልክት አድርጊ							
	ምልክቶች	**ምላሽ**						
		**ውጤት =**	**የለም- ትንሽ- መካከለኛ -ከባድ - በጣም ከባድ**<br>**I-------I---------I --------I-----------I**					
			**0**	**1**	**2**	**3**	**4**	
	ትኩሳትና ማላብ		O	O	O	O	O	T16a
	የልብ አለመርጋት		O	O	O	O	O	T16b
	የእንቅልፍ ችግር		O	O	O	O	O	T16c
	የመደበር ስሜት		O	O	O	O	O	T16d
	መነጫነጭ		O	O	O	O	O	T16e
	ዉጥረት/ጭንቀት		O	O	O	O	O	T16f
	አካላዊና/አእምሮአዊ መድከም/መዛል		O	O	O	O	O	T16g
	የወሲብ ችግር		O	O	O	O	O	T16h
	የፊኛ ችግር		O	O	O	O	O	T16i
	የብልት መድረቅ		O	O	O	O	O	T16j
	የመገጣጠሚያና የጡንቻ ህመም		O	O	O	O	O	T16k
18.	በአንቺ ላይ የታዩት ምልክቶች ጠቅላላ ድምር?		በቁጥር └──┴──┘					T17

ክፍል 3 ፡ ስለ ማረጥ ያለ እውቀት

አሁን ስለማረጥ ክስተት የተለያዩ ጽንሰ ሀሳቦችን የተመለከቱ ጥያቄዎችን እጠይቅሻለሁ			
**ጥያቄ**		**መልስ**	**መለያ**
1.	በማረጥ ወቅት የወርአበባ በድንገት ይቆማል ብለሽ ታስቢያለሽ?	1. አዎ<br>2. አይ	A0
2.	ሴቶች በ48-55 አመታቸዉ ያርጣሉ ብለሽ ታስቢያለሽ?	1. አዎ<br>2. አይ	A1
3.	ከቤተሰብ የሚወረስ የዘር ሁኔታ የማረጥ ሁኔታ ላይ ተፅእኖ ይኖረዋል ብለሽ ታምኛለሽ?	1. አዎ<br>2. አይ	A2
4.	ማረጥ በወሲብ ሆርሞን መጨመር ምክንያት ይከሰታል ብለሽ ታስቢያለሽ?	1. አዎ<br>2. አይ	A3
5.	ቀጭን ሰዎች ቶሎ ያርጣሉ ብለሽ ታምኛለሽ?	1. አዎ<br>2. አይ	A4
6.	ብዙ ሴቶች ከማረጣቸዉ በፊት የወርአበባ መዛባት ያጋጥማቸዋል ብለሽ ታስቢያለሽ?	1. አዎ<br>2. አይ	A5
7.	በማረጥ ወቅት በአብዛኛዉ ሴቶች ትኩሳት እንደሚኖራቸዉ ታዉቂያለሽ?	1. አዎ<br>2. አይ	A6
8.	ማረጥ የብልት ኢንፌክሽን ይቀንሳል ብለሽ ታስቢያለሽ?	1. አዎ<br>2. አይ	A7
9.	ማረጥ ክብደትን ይጨምራል ብለሽ ታምኛለሽ?	1. አዎ<br>2. አይ	A8
10.	የማረጥ ምልክቶችን ማዳንና መቆጣጠር ይቻላል የሚል ስሜት አለሽ?	1. አዎ<br>2. አይ	A9
11.	ማረጥ የልብ ችግሮችን ይቀንሳል ብለሽ ታስቢያለሽ?	1. አዎ<br>2. አይ	A10
12.	ማረጥ የሴቶችን አጥንት መሳሳት እንደሚጨምር ታዉቂያለሽ?	1. አዎ<br>2. አይ	A11
13.	ማረጥ በሴቶች የቆዳ መድረቅና መሟሸሽ ያስከትላል ብለሽ ታስቢያለሽ?	1. አዎ<br>2. አይ	A12
14.	ማረጥ በሴቶች ላይ የተለያዩ የካንሰር አይነቶች እንዲከሰቱ ምክንያት ይሆናል ብለሽ ታስቢያለሽ?	1. አዎ<br>2. አይ	A13
15.	ወሲባዊ ልማዶችና ሁኔታዎች በማረጥ ወቅት ይቀየራሉ ብለሽ ታምኛለሽ?	1. አዎ<br>2. አይ	A14
16.	ማጨስ የማረጫ ጊዜ ላይ ተጽእኖ ያሳድራል ብለሽ ታምኛለሽ?	1. አዎ<br>2. አይ	A15
17.	ማጨስ የማረጥ ምልክቶችን በማወሳሰብና በማክበድ ለተዳዳኝ ህመሞች እንደሚያጋልጥ ታዉቂያለሽ?	1. አዎ<br>2. አይ	A16
18.	ማረጥ በሴቶች ፊት ላይ ያለዉን የፀጉር መጠን የጨምራል ብለሽ ታስቢያለሽ?	1. አዎ<br>2. አይ	A17
19.	ማረጥን የመራቢያ አካል መድረቅንና በወሲብ ጊዜ የህመም ስሜትን እንደሚፈጥር ታዉቂያለሽ?	1. አዎ<br>2. አይ	A18
20.	ማረጥ የሴቶችን የሽንት መሽናት ድግግሞሽ መጨመርና በመሽናት ጊዜ የህመም ስሜት እንደሚፈጥር ታዉቂያለሽ?	1. አዎ<br>2. አይ	A19
21.	ማጨስና አልኮል መጠጦችን መጠጣት የአጥንት መሳሳትን በሴቶች ላይ ያስከትላል ብለሽ ታስቢያለሽ?	1. አዎ<br>2. አይ	A20

22.	ቋሚ የአካል ብቃት እንቅስቃሴ ማድረግ በማረጥ ወቅት የሚመጣን የአጥንት መሳሳት ይከላከላል ብለሽ ታምኛለሽ?	1. አዎ<br>2. አይ	A21
23.	ማረጥ የሴቶችን የትኩረትና የማስታወስ አቅም ተጽእኖ እንደሚያደርግ ታዉቂያለሽ?	1. አዎ<br>2. አይ	A22
24.	የሚያርጡ ሴቶች ከወትሮዉ በተለየ ሁኔታ እየጨመረ የሚመጣ ሰዉነትን የማላብና ሙቀት መጨመር ይከሰታል ብለሽ ታስቢያለሽ?	1. አዎ<br>2. አይ	A23
25.	ባረጠች ሴት ላይ የድብርትና የጭንቀት መጠን ከወትሮዉ ይጨምራል ብለሽ ታምኛለሽ?	1. አዎ<br>2. አይ	A24
26.	የወርአበባ በቆመ ከአንድ አመት በኋላ እርግዝና መከላከያ መንገዶችን መጠቀም ያስፈልጋል ብለሽ ታምኛለሽ?	1. አዎ<br>2. አይ	A25

ክፍል 4 ፡ ስለ ማረጥ ያለአመለካከት

አሁን ስለማረጥ ከስተት ያለሽን አመለካከት የተመለከቱ ጥያቄዎችን እጠይቅሻለሁ			
**ጥያቄ**		**መልስ**	**መለያ**
1.	ማረጥ ለሴቶች የብቸኝነት ወቅት ነዉ	1. ሙሉበሙሉ እስማማለሁ<br>2. እስማማለሁ<br>3. አልስማማም<br>4. ሙሉበሙሉ አልስማማም	P0
2.	ማረጥ ለሴቶች የወርአበባ ጭንቀትንና እርግዝናን የሚከላከሉበት ወቅት ነዉ	1. በጣም እስማማለሁ<br>2. እስማማለሁ<br>3. አልስማማም<br>4. በጣም አልስማማም	P1
3.	ማረጥ የባሎችን ወሲባዊ ፍላጎት ይቀንሳል	1. በጣም እስማማለሁ<br>2. እስማማለሁ<br>3. አልስማማም<br>4. በጣም አልስማማም	P2
4.	ሁሉም ሴቶች አካላዊ እንቅስቃሴ በማድረግ ለእራሳቸዉ አስፈላጊዉን እንክብከቤ ማድረግ ይችላሉ	1. በጣም እስማማለሁ<br>2. እስማማለሁ<br>3. አልስማማም<br>4. በጣም አልስማማም	P3
5.	በማረጥ ወቅት ለባሎች ያለዉ ፍላጎትና የሚደረገዉ ትኩረት ይቀንሳል	1. በጣም እስማማለሁ<br>2. እስማማለሁ<br>3. አልስማማም<br>4. በጣም አልስማማም	P4
6.	ማረጥ የሴቶች አቅምና ጉልበት መድከም የሚጀምርበት ወቅት ነዉ	1. በጣም እስማማለሁ<br>2. እስማማለሁ<br>3. አልስማማም<br>4. በጣም አልስማማም	P5
7.	ሴቶች ካረጡ በኋላ ህይወታቸዉ ከማረጣቸዉ በፊት ከነበረዉ የበለጠ ደስተኛ ይሆናል	1. በጣም እስማማለሁ<br>2. እስማማለሁ<br>3. አልስማማም<br>4. በጣም አልስማማም	P6
8.	ማረጥ የአንድን ሴት አካላዊ ዉበት ይቀንሰዋል	1. በጣም እስማማለሁ	P7

		2. እስማማለሁ<br>3. አልስማማም<br>4. በጣም አልስማማም	
9.	ማረጥ በሴቶች ህይወት ዉስጥ የሚከሰት ተፈጥሮአዊ ሁኔታ ነዉ	1. በጣም እስማማለሁ<br>2. እስማማለሁ<br>3. አልስማማም<br>4. በጣም አልስማማም	P8
10.	ማረጥ አዲስ ህይወት የመጀመሪያና የሴቶች ሌላ የህይወት ምእራፍ ነዉ	1. በጣም እስማማለሁ<br>2. እስማማለሁ<br>3. አልስማማም<br>4. በጣም አልስማማም	P9

ክፍል 5 ፡ ስለ ማረጥ ያለ እውቀት እና አመለካከትን ተጽእኖ የሚያደርጉ

አሁን የሴቶችን በማረጥ ላይ ያላቸዉን እዉቀትና አመለካከት ተጽእኖ አድራጊዎችን በተመለከተ የተወሰኑ ጥያቄዎችን እጠይቅሻለሁ፡፡ እነሱም ስለማረጥ ከሌሎች ሰዎች ጋር ያለፍርሀት ማዉራት፣ አካላዊ ጤንነት፣ የስሜት ደህንነት፣ የመረጃ ምንጮች፣ ስለማረጥ ያለፍርሀት የሚያዋዩዋቸዉ የጓደኞችና የቤተሰብ አባላት ብዛት፡፡			
**ጥያቄ**		**መልስ**	**መለያ**
1.	ስለ ማረጥ መረጃዎችን ሰምተሽ/ወይም አንብበሽ ታዉቂያለሽ	1. አዎ<br>2. አይ-------------**ወደ Q2 እለፊ**	Q0
2.	መልስሽ አዎ ከሆነ መረጃዎችን ያገኘሽዉ ምንጩ ከየት ነዉ?	1. ከጓደኛ	Q1a
		2. ከህክምና አገልግሎት ሰጪዎች	Q1b
		3. መጻህፍት፣መጽሔቶች፣ጆርናሎች	Q1c
		4. መገናኛ ብዙሀን(ራድዮ፣ቴሌቪዥን)	Q1d
		5. ከዘመዶች	Q1e
		6. ከትምህርታዊ ፕሮግራሞች	Q1f
		7. ከጓደኛ	Q1g
		8. ከህክምና አገልግሎት ሰጪዎች	Q1h
3.	ከሌሎች ሰዎች ጋር ስለ ማረጥ ማውራት ?	1. በጣም ቀላል ነው<br>2. ቀላል ነው<br>3. ከባድ ነው<br>4. በጣም ከባድ ነው	Q2
4.	ስለ ማረጥ ያለፍርሀት የምታወሪያቸው ምን ያህል ጓደኖች አሉሽ?	በቁጥር |__|__|	Q3
5.	ስለ ማረጥ ያለፍርሀት የምታወሪያቸው ምን ያህል ቤተሰቦች አሉሽ?	በቁጥር |__|__|	Q4
6.	በአሁን ሰአት ያለሽበትን የሥሜት ሁኔታ እንዴት ትገልጪዋለሽ	1. በጣም ደስ የማይል<br>2. ደስ የማይል<br>3. በጣም ጥሩ<br>4. እጅግ በጣም ጥሩ	Q5
7.	አውን ያለሽበትን አካላዊ ጤና እንዴት ትገልጪዋለሽ	1. በጣም ህመምተኛ<br>2. ህመምተኛ<br>3. ደህና<br>4. በጣም ጤነኛ<br>5. እጅግ በጣም ጤነኛ	Q6
